# Immunization with DNA prime-subunit protein boost strategy based on influenza H9N2 virus conserved matrix protein M1 and its epitope screening

**DOI:** 10.1038/s41598-020-60783-z

**Published:** 2020-03-05

**Authors:** Fen Liu, Xueliang Wang, Mei Zheng, Feifei Xiong, Xueying Liu, Linting Zhou, Wensong Tan, Ze Chen

**Affiliations:** 1 Shanghai Institute of Biological Products, Shanghai, 200052 China; 2 State Key Laboratory of Bioreactor Engineering, East China University of Science and Technology, Shanghai, 200237 China; 3 Department of Molecular Biology, Shanghai Centre for Clinical Laboratory, Shanghai, China

**Keywords:** DNA vaccines, Protein vaccines

## Abstract

Developing an effective universal influenza vaccine against influenza virus with highly conserved antigenic epitopes could induce a broad-spectrum immune response to prevent infection. The soluble protein M1 that can induce the M1 specific immune response was first confirmed in our previous study. In this study, we characterized the immune response induced by DNA prime-subunit protein boost strategy based on the relatively conserved matrix protein 1 (M1) in the BALB/c mouse model, and evaluated its protection ability against a lethal challenge of homologous H9N2 avian influenza virus (A/Chicken/Jiangsu/11/2002). The results showed that 100 μg DNA prime + 100 μg M1 subunit protein boost-strategy significantly increased antibody levels more than vaccination with M1 DNA or M1 subunit protein alone, and induced a more balanced Th1 / Th2 immune response, which not only can provide protection against the homologous virus but also can provide part of the cross-protection against the heterosubtypic PR8 H1N1 strain. In addition, we used an Elispot assay to preliminary screen the T cell epitope in M1 protein, and identified that p22 (M1_11–25_ VLSIIPSGPLKAEIA) epitope was the only immunodominant M1-specific CD4^+^ T cell epitopes, which could be helpful in understanding the function of influenza virus T cell epitopes.

## Introduction

Vaccination is the most effective way to prevent influenza virus infection^1,2^. Current influenza vaccines are based on induction of protective antibodies against the viral surface hemagglutinin (HA) protein, which can effectively neutralize the influenza virus and significantly reduce morbidity and mortality. However, owing the variability of HA, vaccine strains need to be changed every year^3–5^.Vaccines based on conserved antigens would not require prediction of which strains are likely to circulate during an approaching season and could avoid hurried manufacturing in response to outbreaks^6,7^. Therefore, development of a universal vaccine has become a research focus.

Matrix protein 1 (M1) is a conserved influenza virus antigen. M1 is a multifunctional protein which plays an important role in virus replication^8–10^. Recently, some groups have developed broad-spectrum vaccines based on the M1 protein of influenza A virus. Okuda *et al*. found that an M protein-based DNA vaccine provided protection against challenge with homologous and heterologous influenza virus^5^. Our previous study demonstrated that DNA vaccines encoding M1 provided mice with partial protection against challenge with homologous influenza virus^11^. In addition, our study first confirmed that mucosal immunization with soluble protein M1 adjuvanted with chitosan could induce an M1-specific immune responses in experimental animals, providing full protection against homologous H9N2 virus challenge as well as partial protection against heterologous H5N1 and PR8 virus challenge^12^. However, due to the immunogenicity of DNA or soluble protein vaccines are not very effective, they needed to be immunized many times or in combination with adjuvants to enhance immune responses.

Wang *et al*. reported that DNA vaccines encoding different H5 HAs could elicit varying degrees of broad-spectrum immune responses prior to the influenza pandemic. Protective antibodies could thus be induced robustly following boosting with inactivated H5 avian influenza vaccine in the event of a pandemic^13^. Plasmid DNA vaccines as stimulus can effectively enhance the immunogenicity of protein vaccines, so the DNA prime - protein boost strategy can enhance cellular and humoral immune responses. Many animal experiments confirmed that the prime-boost strategy can effectively protect against a variety of bacteria and protozoan pathogens^14–18^. Wei *et al*. used the HA DNA prime-trivalent inactivated vaccine (TIV) boost strategy to induce high titres of neutralization antibodies and cross-reactive immune responses^19^.

In this study, a DNA plasmid encoding M1 and soluble recombinant M1 protein from an avian H9N2 isolated strain (A/Chicken/Jiangsu/11/2002) were used to immunize BALB/c mice using a DNA prime-protein boost strategy. Immune responses and the protection in a mouse model were evaluated. We showed that a 100 μg DNA prime +100 μg M1 subunit protein boost strategy can effectively induce cellular and humoral immune responses and provide complete protection against homologous virus infection as well as partial cross-protection against heterosubtypic virus infection.

## Results

### Protection against lethal avian influenza H9N2 virus challenge in mice by DNA prime intranasal protein boost strategy based on M1 vaccine

Female BALB/c mice (n = 152) were randomly divided into eight groups, with 19 mice per group. Mice were immunized as described in the methods section. Two weeks after the last immunization, all mice were intranasally challenged with a lethal dose (20 × LD_50_) of A/Chicken/Jiangsu/7/2002 (H9N2) virus. On days 3, 5 and 7 following the lethal challenge, three randomly selected mice from each group were acrificed. The lung homogenate and bronchoalveolar lavage fluids (BALF) was collected and used for virus detection (Table 1, Table S1). The survival rates and the body weight losses of the remaining 10 mice in each group were monitored for 21 days post-challenge to evaluate protection against the homologous A/Chicken/Jiangsu/7/2002(H9N2) virus.

**Table 1:** Protection against lethal avian influenza H9N2 virus challenge in mice by DNA prime intranasal protein boost strategy based on M1 vaccine.

Group	Immunogen	Lung virus titer (log_10_TCID_50_/ml)^a^			Survival rate (No. Of survivors/no. tested)
		3 days	5 days	7 days	
A	100 μg DNA+100 μg M1	5.25 ± 0.25	4.08 ± 0.28^b^	1.92 ± 0.14^b,c^	10/10^b,c^
B	100 μg DNA+10 μg M1	5.58 ± 0.72	5.08 ± 0.38	3.58 ± 0.52^b,d^	1/10
C	100 μg DNA+1 μg M1	5.41 ± 0.38	4.83 ± 0.14	4.16 ± 0.14	0/10
D	100 μg M1	5.00 ± 0.00	3.91 ± 0.14^b^	3.08 ± 0.14^b^	4/10^b^
E	10 μg M1	5.83 ± 0.14	4.67 ± 0.14	4.58 ± 0.14	0/10
F	1 μg M1	5.67 ± 0.52	5.08 ± 0.14	4.67 ± 0.14	0/10
G	100 μg DNA	5.58 ± 0.14	5.00 ± 0.00	4.42 ± 0.14	0/10
H	control	5.75 ± 0.25	5.33 ± 0.38	4.58 ± 0.14	0/10

One hundred and fifty-two BALB/c mice were randomly divided into groups of eight. Nineteen mice in each group were immunized as described above. Two weeks post-vaccination, mice were challenged with a lethal dose (20 × LD50) of avian influenza H9N2 virus (homologous virus). BALF from three mice in each group were collected on the 3rd day, 5th day and 7th day post-infection for titration of lung virus respectively. The survival rate of mice 21 days post-infection was determined.

^a^Results are expressed as mean ± SD of tested mice in each group.

^b^Significant differences compared to the mice in control group (p < 0.05).

^c^Significant differences compared to the mice in D group (p < 0.05).

^d^Significant differences compared to the mice in E group (p < 0.05).

The results in Table 1 showed that the protection of mice immunized with 100 μg (Group D), 10 μg (Group E) and 1 μg (Group F) of M1 protein alone were 40% (4/10), 0% (0/10) and 0% (0/10), respectively. The protection of mice immunized with 100 μg DNA+100 μg M1 protein (Group A, DNA+100 μg), 100 μg DNA+10 μg M1 protein (Group B, DNA+10 μg) and 100 μg DNA+1 μg M1 protein (Group C, DNA+1 μg) were 100% (10/10), 10% (1/10) and 0% (0/10), respectively. These results suggested that the better protection of mice in DNA prime + M1 protein intranasal boost groups were better than that in M1 protein alone groups, and that protection of mice required a high dosage of M1 protein. Mice that received DNA vaccine alone (Group G) and control mice (Group H) had all died within 10 days of challenge. Thus, compared with the DNA vaccines alone or the recombinant M1 subunit vaccine alone, the DNA prime-M1 protein intranasal boost strategy could effectively improve the protection effect of vaccines.

Following challenge, all mice exhibited obvious viral infection symptoms. To evaluate changes in viral abundance in the lung following immunization, we collected the BALF at 3, 5 and 7 days after the challenge to quantitate viral titres (Table 1). Three days post-challenge, the lung viral titres of all immunized mice is high (> 5 log_10_TCID_50_/ml), and did not differ significantly from those of the control group (P > 0.05). Thus, the mice likely lacked enough specific neutralizing antibodies to resist effectively early infection and replication of the influenza virus. At 5 days post-challenge, the virus titres in Group A (DNA+100 μg M1 protein) were significantly lower than those observed on day 3 (P < 0.05). Viral titres in all other groups reached their peak on day5 post-challenge. On the 7th day post-challenge, the viral titres in almost all mice began to fall. Viral titres in the lungs of mice in group A were significantly lower than those of the control group (H) and the corresponding dose of M1 protein alone immune group (Group D) (P < 0.05). Moreover, the lung viral titres of the mice in group A showed the greatest reduction (<2 log_10_TCID_50_/ml). These results showed that the DNA prime + M1 protein boost immune strategy could provide enhanced protection compared with the corresponding dose of M1 protein alone. Among mice immunized with the prime-boost strategy, the virus clearance ability of mice in Group A was significantly better than group B and group C. Among mice immunized with M1 protein alone, the virus clearance ability of mice in group D was better than group E and group F. These data suggested the M1 protein immunization dose was positively correlated with virus clearance ability.

**Figure 1:**
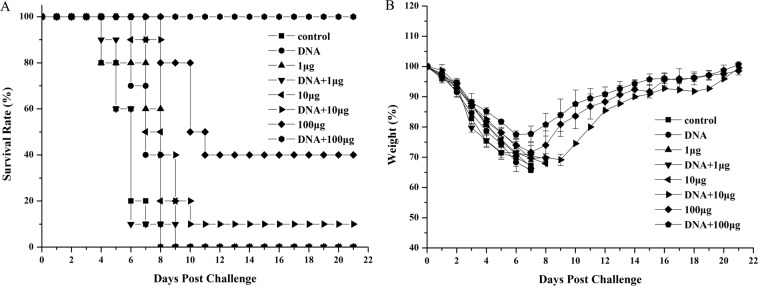
Survival rates (**A**) and body weight changes (**B**) after a challenge with lethal homologous influenza virus. Mice were immunized as described above. Two weeks post-vaccination, mice were challenged with a lethal dose (20 × LD_50_) of the mouse-adapted A/Chicken/Jiangsu/7/2002 (H9N2) virus. The survival rates and body weights of the mice were measured daily from the date of the challenge to 21 days after the challenge. Values represent means ± SD of each group of mice.

The body weights of mice were monitored following challenge (Fig. 1B). All mice lost weight after the challenge, but the mice in group A had the smallest weight loss and were restored fastest. Live mice in groups A, B and D began to recover on the 8th day post-challenge, whereas the mice in other groups all died within 10 days.

### Serum and mucosal antibody response in mice induced by DNA prime-intranasal protein boost strategy based on M1

Eight groups of mice were immunized respectively according to the materials and methods section. Two weeks after the last immunization, serum IgG, nasal wash IgA and IgA antibody levels in BALF were tested. As shown in Table 2, compared with the control group (Group H), specific IgG antibody could be detected in all experimental mice. The specific antibody levels in mice immunized with DNA+100 μg M1 protein (Group A) were significantly higher than in mice immunized with the corresponding dose of M1 protein alone immune group (P < 0.05). We found that IgG antibody tites increased with increasing protein doses. Nasal wash IgA antibodies levels in mice showed that, compared to the control group, only the DNA+100 μg M1 protein (Group A) can significantly detect the IgA antibodies (P < 0.05), other immune groups could not detect IgA antibodies. At the same time, we also tested BALF IgA antibody levels in mice and found that they were slightly higher than those in the nasal wash, and IgA antibody titers increased with the increase of doses of protein. The BALF IgA antibody titers in mice of the Group A were significantly higher than that in Group B and the corresponding dose of M1 protein alone immune group (Group D) (P<0.05). These data showed that DNA primary - intranasal protein boost strategy based on M1 could effectively enhance the humoral immune response.

**Table 2:** Serum and mucosal antibody responses in mice induced by prime-boost strategy based on M1^a^.

Group	Immunogen	Serum IgG (ELISA,2^n^)	Mucosal IgA (ELISA,2^n^)		IgG subclasses (ELISA,2^n^)		IgG1/IgG2a ratio
			Nasal wash IgA	BALF IgA	IgG1	IgG2a	
A	100 μg DNA+100 μg M1	13.67 ± 2.09^b,c,d,g^	1.33 ± 0.58	6.33 ± 1.15^b,d^	15.00 ± 2.00^c,g^	14.00 ± 1.00^b,c,d,g^	1.07
B	100 μg DNA+10 μg M1	11.67 ± 1.15^e,g^	ND^h^	1.33 ± 0.58	12.67 ± 0.58^e,g^	12.00 ± 1.00^e,g^	1.06
C	100 μg DNA+1 μg M1	10.67 ± 0.58 ^f,g^	ND^h^	ND^h^	10.33 ± 0.58 ^f,g^	9.00 ± 1.00 ^f,g^	1.15
D	100 μg M1	11.00 ± 1.00^e,f^	ND^h^	3.67 ± 1.15	12.67 ± 1.15^e,f^	6.67 ± 0.58^e,f^	1.90
E	10 μg M1	8.33 ± 1.53	ND^h^	1.00 ± 0.00	9.67 ± 1.53	3.67 ± 1.15	2.64
F	1 μg M1	5.33 ± 0.58	ND^h^	ND^h^	4.33 ± 1.15	2.33 + 0.57	1.86
G	100 μg DNA	7.33 ± 0.58	ND^h^	ND^h^	7.33 ± 1.53	7.33 ± 0.58	1.00
H	control	—	—	—	—	—	—

Mice were immunized as described above. Two weeks post-vaccination, serum, nasal wash, and BALF of three mice in each group were prepared and examined by ELISA for M1 specific IgG, IgA, IgG1 and IgG2a Abs, respectively.

^a^Results are expressed as mean ± SD of three tested mice in each group.

^b^Significant differences compared to the mice in B group (p < 0.05).

^c^Significant differences compared to the mice in C group (p < 0.05).

^d^Significant differences compared to the mice in Dgroup (p < 0.05).

^e^Significant differences compared to the mice in E group (p < 0.05).

^f^Significant differences compared to the mice in F group (p < 0.05).

^g^Significant differences compared to the mice in G group (p < 0.05).

^h^ND. not detected.

In mice, IgG2a is related to the Th1 immune response, and IgG1 is related to the Th2 immune response. To further characterize the humoral immune responses of mice, we tested IgG1 and IgG2a antibody subclass levels and calculated their ratio (IgG1/IgG2a) in different immunization groups. As shown in Table 2, the prime-boost immunization groups (Group A, B and C) had high IgG1 antibody titres similar to the corresponding protein alone groups (Group D, E and F). However, the prime-boost immunization groups also had higher IgG2a antibody than the protein alone groups. The IgG1/IgG2a ratios was closer to 1 in the prime-boost groups, indicating that the prime-boost strategy could effectively enhance Th1 immune responses resulting in a balanced Th1/Th2 immune response. By contrast, immunization with protein alone induced higher IgG1 and the ratios >1.8, reflective of a Th2 bias.

**Figure 2:**
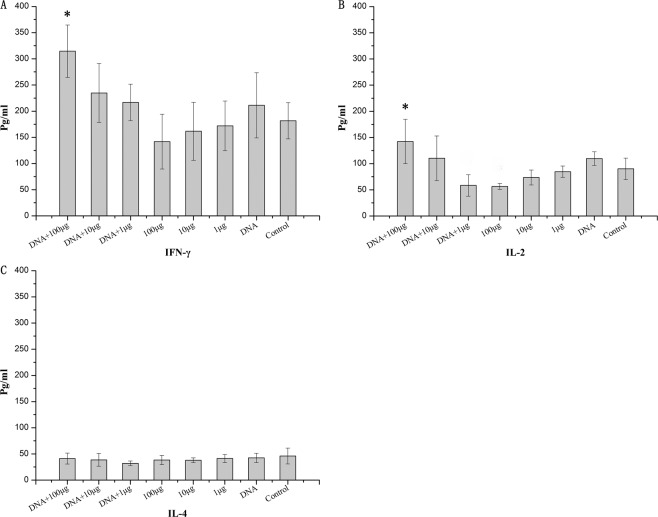
The expression of cytokines in the lungs. Mice were immunized as described above. Two weeks after the last administration, mice were challenged with a lethal dose (20 × LD_50_) of the mouse-adapted A/Chicken/Jiangsu/7/2002 (H9N2) virus. The BALFs were collected and the selected cytokines were quantitatively analyzed. Panels A, B and C represent data obtained from measurements of IFN-γ, IL-2, and IL-4, respectively. *Significant differences compared to the corresponding M1 protein immunization group alone (p < 0.05).

To further confirm the above hypothesis, we detected cytokines in the BALF to monitor levels of cytokine levels such as IL-4, IFN-γ and IL-2 in the lung. Levels of IL-2 and IFN-γ in group A were significantly different compared with mice immunized with the corresponding dose of M1 protein alone (P < 0.05) (Fig. 2A,B). This was consistent with the previous result showing that group A induced the highest IgG2a antibody response which may be induced by type 1 cytokines (Table 2). We found that levels of IL-4 did not differ between each group (Fig. 2C), indicating that the prime-boost strategy did not effect Th2 type cellular immune responses. This finding was also consistent with the IgG1 antibody response (Table 2).

### Cellular immune response in mice induced by DNA prime-intranasal protein boost strategy based on M1

To further confirm the enhancement effect of Th1 type cellular immune responses by the prime-boost immune strategy, we used M1 peptide pools as the stimulus in ELISPOT assays to test the IFN-γ secretion of T lymphocytes from immunized mice. Twenty-four female BALB/c mice aged 6 to 8 weeks were divided into 8 groups of three mice each. Two weeks after the last immunization, the mice were sacrificed, and spleens were isolated to quantitate the IFN-γ secreting T cells.

**Figure 3:**
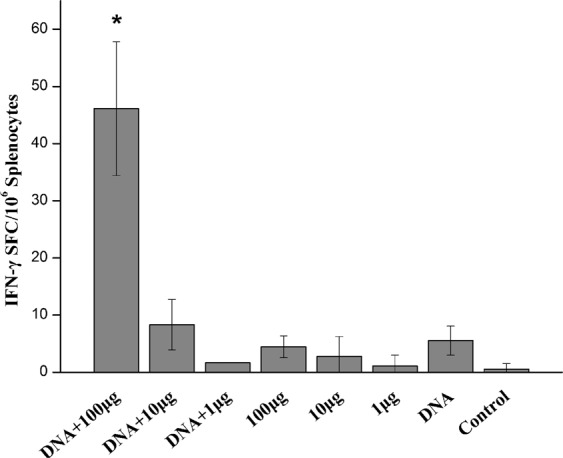
Mice primed with 100 μg DNA-boosted with 100 μg M1 protein generated more M1 specific IFN-γ producing T cells compared with mice immunized alone. Splenocytes harvested from mice 14 days after vaccination were stimulated with 50 μg/ml M1 pools for 22-24 h and scored in ELISPOT assays for IFN-γ producing cells. The values represent the averages of quadruplicate wells of 3 mice, and are expressed as means ± SD. The results were expressed as the number of SFC per 10^6^ input cells. *Significant differences between the treatment groups and the control group (p < 0.05).

Quantitation of specific IFN-γ spots (Fig. 3) showed that the highest number of specific IFN-γ spots appeared in the spleens of mice from the DNA+100 μg M1 protein group, with very few specific spots observed in the other groups. These results showed that the DNA prime-intranasal protein boost immunization strategy could significantly enhance the capacity of vaccines to induce cellular immune responses.

### Heterosubtypic protection against lethal PR8 influenza virus in mice by DNA prime intranasal protein boost strategy based on M1 vaccine

**Figure 4:**
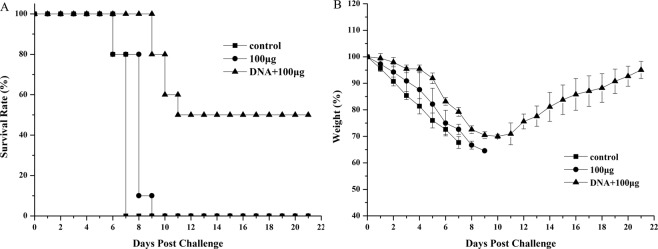
Survival rates (**A**) and body weight changes (**B**) after challenge with lethal heterosubtypic influenza virus. Mice were immunized as described above. Two weeks after the last immunization, mice were challenged with a lethal dose (5 × LD_50_) of mouse-adapted heterosubtypic PR8(H1N1) influenza A virus. The survival rates and body weights of the mice were measured daily from the date of the challenge to 21 days after the challenge. Values represent means ± SD of each group of mice.

Owing to the fact that only the mice in DNA+100 μg M1 protein immunized group were completely protected against lethal challenge with homologous virus, we used this group of mice to evaluate cross protection. Unimmunized mice and mice immunized with the corresponding protein alone were used as controls. Thirty-nine mice were randomly divided into 3 groups, with 13 in each. Two weeks after the last immunization, the mice were intranasally challenged with 5 × LD_50_ A/Puerto Rico / 8/34 (H1N1) (PR8) and then observed for 21 days to assess mortality and weight changes (Table 3). Following challenge, the 3 groups of mice all appeared to have obvious infection symptoms, of which the infection symptoms of mice in the unimmunized group and protein alone group were serious and all died on the 10th to 14th days, respectively. Although mice in the prime-boost group also showed severe symptoms, 50% of them survived (Fig. 4A), and the body weights of surviving mice began to gradually recover from the 10^th^ day post-challenge and reached baseline on the 21^st^ day post-challenge (Fig. 4B). Thus, compared with the protein alone group, the DNA prime-protein boost immune strategy could provide partial protection against lethal heterosubtypic influenza virus challenge.

**Table 3:** Heterosubtypic protection against lethal PR8 influenza virus in mice by prime-boost strategy based on M1 vaccine.

Group	Immunogen	Survival rate (No. Of survivors/no. tested)
A	100 μg DNA+100 μg M1	5/10^a^
D	100 μg M1	0/10
H	Control	0/10

Mice of three groups were immunized as described above. Two weeks after the last immunization, mice were challenged with a lethal dose (5 × LD50) of influenza heterosubtypic virus PR8. The survival rate of mice 21 days post-infection was determined.

^a^Significant differences compared to the mice in control group (p < 0.05).

### Overall M1-specific T cell response ***in vitro***

**Figure 5:**
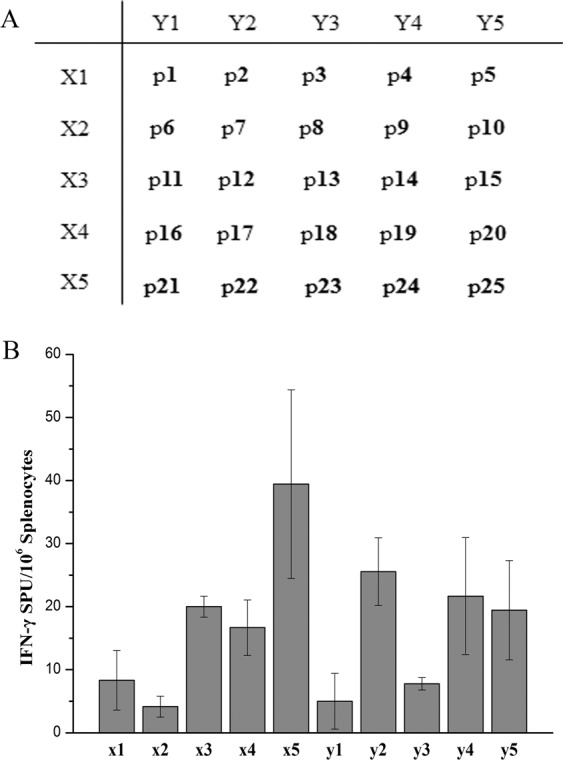
T cell epitope screening of peptide library spanning the entire M1 protein of H9N2 avian influenza virus. (**A**) A library of overlapping peptides spanning the entire M1 protein divided into ten pools of 5 peptides each according to a 5 × 5 matrix. 25 peptides were randomly named with number 1 to 25 and listed according to the matrix above. Peptides in the same line or column mixed as one pool, respectively. Then each pool as a stimulus for ELISPOT assay. (**B**) Peptide pool detection. Two weeks after the DNA primary - 100 μg M1 intranasal boost immune strategy, the spleen lymphocytes were isolated, 2 × 10^5^ responding cells were incubated with each pool (50μg/ml) for 20-24 hrs in 96-well PVDF plates coated with anti-IFN-γ monoclonal antibody, then calculated the specific spots. The spots >10 were as the positive peptide pools. The values represent the averages of quadruplicate wells of 3 mice, and are expressed as means ± SD. The results were expressed as the number of SFC per 10^6^ input cells.

**Figure 6:**
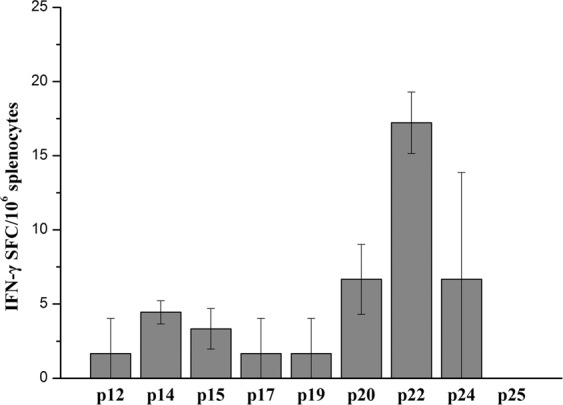
Peptide activity of M1 T cell epitope of H9N2 avian influenza virus. Two weeks after the DNA primary - 100 μg M1 intranasal boost immune strategy, the spleen lymphocytes were isolated, 2 × 10^5^ responding cells were incubated in 96-well PVDF plates coated with anti-IFN-γ monoclonal antibody. According to a 5 × 5 matrix, the single peptide of the intersection of two positive peptide pools may be the M1 specific T cell epitope peptide, the preliminarily determined positive single peptides were respectively used as ELISPOT reaction stimulus (50 μg/ml peptide), then to calculate the specific spots after 20-24 hrs. The peptide with spots >10 was considered positive peptides. The values represent the averages of quadruplicate wells of 3 mice, and are expressed as means ± SD. The results were expressed as the number of SFC per 10^6^ input cells.

To further understand the M1 specific immune response, we sought to identify T cell epitopes within the M1 protein. We prepared an M1 peptide library including 25 peptides. According to the a 5 × 5 matrix (Fig. 5A), two series of peptide pools X and Y were designed. We ensured that each unique peptide existed in the X or Y peptide pool. These 10 peptide pools were used to assess spleen cell reactivity by ELISPOT (DNA+100 μg M1 protein immune group). As shown in Fig. 5, following prime-boost immune, T cells from BALB/c mice (H-2^d^) could recognize 6 peptide pools (spot frequency >10 was considered positive): X3, X4, X5, Y2, Y4 and Y5. We also identified 9 common single peptides (p12, p14, p15, p17, p19, p20, p22, p24 and p25), which may contain in one or more M1 specific T cell epitopes. We analyzed and compared the ability of nine peptides to stimulate the T cells of BALB/c mice (H-2^d^) to secrete IFN-γ. As shown in Fig. 6, p22 could obviously stimulate T cells secreting IFN-γ, while p20 and p24 had weaker effects.

**Figure 7:**
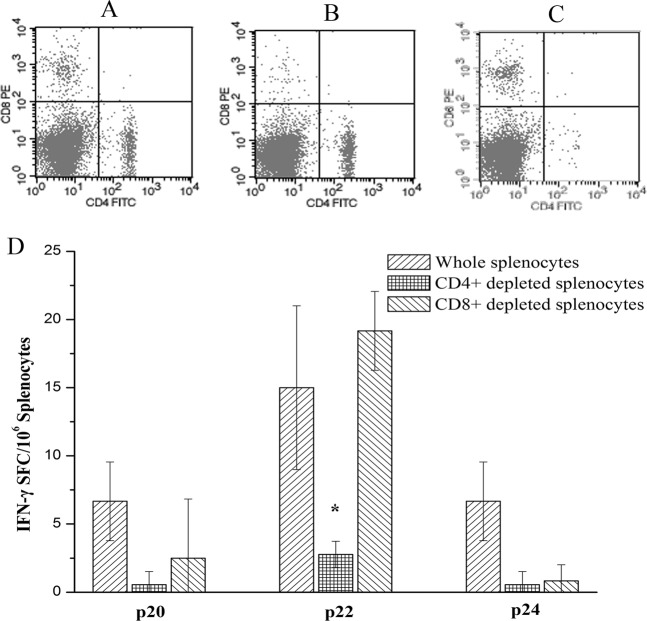
CD4^+^T/CD8^+^T cell depletion ELISPOT assay. (1) CD4^+^T/CD8^+^T cell depletion. Two weeks after the DNA primary - 100 μg M1 intranasal boost immune strategy, the spleen lymphocytes were isolated and CD8 ^+^ T and CD4 ^+^ T cells were removed respectively by using MACs microbeads. (**A**) before the mixed lymphocyte sorting; (**B**) after removing the CD8^+^ T cells; (**C**) after removing the CD4^+^ T cells. (2) Collecting the MACs positive sorting cells. 2 × 10^5^ responding cells were incubated in 96-well PVDF plates coated with anti-IFN-γ monoclonal antibodies, the single peptide p22 was used to stimulate three kinds of different cells respectively, and the weakly positive single peptide p20 and p24 were used as the control (50 μg/ml peptide). (**D**) left-slash representing the whole spleen lymphocytes, cross hatch representing the spleen lymphocytes removing CD4^+^ T cells, right-slash representing the spleen lymphocytes removing CD8^+^ T cells. The values represent the averages of quadruplicate wells of 3 mice, and are expressed as means ± SD. The results were expressed as the number of SFC per 10^6^ input cells. *Significant differences compared to the mice in the whole splenocytes group (p < 0.05).

To further characterize p22 peptide reactivity, we obtained CD4^+^ CD8^−^ and CD4^−^CD8^+^ splenic T cell using MACs microbeads (Fig. 7). Both types of lymphocytes were used for ELISPOT analysis *in vitro* with p22 as the stimulus. As shown in Fig. 7D, the control peptides p20 and p24 could not effectively stimulate the corresponding lymphocytes. By contrast, p22 can stimulate both the CD4^+^ CD8^+^ T and CD4^+^ CD8^−^ splenic T cells to secret IFN-γ. The p22 could not effectively stimulate CD4^−^CD8^+^ splenic T cell to secret IFN-γ, suggesting that p22 (M1_11-25_ VLSIIPSGPLKAEIA) could be a CD4^+^ T cell epitope.

## Discussion

The influenza virus crosses the species barrier to threaten human health and safety^20^. Examples include the recent emergence of H5N1 avian influenza virus^21^, the H1N1 swine influenza virus^22^ and the H7N9 avian influenza virus^23^, and therefore vaccination is very important. The antibody response generated by traditional seasonal vaccines can only neutralize matching influenza virus strains, but cannot confer effective protection against antigenically mutanted strains or emerging epidemic strains^24^. A universal influenza vaccine is expected to provide protection in a new way^25^. The universal influenza vaccines over the past several decades^26^ have focused on targeting the conserved M and NP proteins^5,27–29^. Influenza vaccines based on the M and NP can induce broad-spectrum anti-viral protection against heterosubtypic influenza virus^6,30^. Our previous studies confirmed that the M1 protein can be used as a candidate for universal vaccines, and that soluble M1 protein adjuvanted with cholera toxin (CT) can induce significant protective effect against heterosubtypic influenza virus challenge^12^. However, the control group immunized without adjuvant was not effectively protected. To improve the effectiveness of this vaccine, this study adopted a prime-boost immunization strategy with M1 vaccine to elicit protective immunity in the absence of an adjuvant. Compared with single immunization, prime-boost immunization strategies can induce an enhanced humoral and cellular immune responses. This strategy provided complete protection against homologous influenza virus challenge as well as partial cross protection against heterosubtypic influenza virus challenge.

In this study, the mice were immunized with a DNA prime-subunit protein boost strategy based on M1, and found the mice in 100 μg DNA primary + 100 μg M1 protein boost group can achieve full protection, whereas the mice in 100 μg M1 protein alone group can achieve a 40% protective effect with a homologous virus challenge. As shown in Table 2, compared with the DNA alone and protein alone vaccine groups, the IgG antibody levels in the joint vaccine immunization group increased significantly compared with the corresponding single vaccine immunization groups. In particular, IgG2a subtype antibody levels were significantly enhanced. These results showed that immunization with protein alone (Group D) induced higher titres of IgG1 antibodies, whereas prime-boost immune groups (Group A) induced higher IgG2a antibody titres. Thus, the prime-boost strategy might effectively enhance Th1 immune responses, resulting in a balanced Th1/Th2 immune response. IFN-γ and IL-2 are generally associated with Th1 type cellular immune responses and can stimulate IgG2a production. IL-4 levels are generally associated with Th2 type cellular immune responses and can induce IgG1 and IgE production. We further found that levels of IL-4 did not differ between groups (Fig. 2C), and that IL-2 and IFN-γlevels in group A were significantly different compared with mice immunized with the corresponding dose of M1 protein alone (P < 0.05) (Fig. 2A,B). This is consistent with the fact that group A mice had the highest IgG2a antibody responses which may be induced by type 1 cytokines (Table 2). Thus, that low IL-4 levels in prime-boost groups similar to control group, suggesting this strategy did not effect Th2 type cellular immune responses. These results are also consistent with the IgG1 antibody response data (Table 2). IgG2a antibody can induce NK cell mediated cytotoxic effect and regulate macrophages^31,32^, so we speculated that M1 specific non-neutralizing antibody IgG2a may play a partial role in virus clearance in the prime-boost group. In the mouse model, compared with Th2 type biased immune responses, Th1-biased or balanced immune responses are more effective in clearing the virus^33,34^. The lung viral titres of mice in the prime-boost decreased quickly, in agreement with this idea. The results of the ELISPOT experiment also confirmed that the prime-boost strategy could induce cellular immune response (Fig. 3). Many studies have confirmed that cell-mediated immune responses are important for removal of influenza A virus^35^.

IgA antibodies as the first immune defense line of mucous membranes on the surface, through the combination with pathogens, prevent pathogens from adsorbing host cells and can further effectively inhibit proliferation and infection by pathogens^36,37^. Price *et al*.^29^ used an adenoviral vector to build a universal influenza vaccine and confirmed that the mucosal immunization could not only induce IgG antibody responses and extensive mucosal antibody, but also remove the virus more quickly and provide more durable protection. To induce the mucosal antibody responses, mice were intranasally immunized with soluble M1 protein. The prime- boost strategy could induce IgG antibodies and IgA antibodies at higher levels compared with the vaccine alone group (Table 2). In previous studies, specific IgA antibody levels were positively correlated with survival rates of mice, which was consistent with our previous experiment^12^. A potential mechanism might involve the action of IgA antibodies on infected epithelial cells, thus affecting virus assembly by interfering with the function of newly synthesized M1 protein^12,38^. In our study, the mice in group A showed 100% homologous virus protection and 50% heterosubtypic virus protection following immunization. We also detected M1-specific IgA antibodies after immunization. In combination with the above studies, this suggested that mucosal antibodies had a role in conferring anti-viral protection.

It is important to note that not all prime-boost schemes can induce significantly enhanced immune responses^39^. In our study, only boosting with high doses of protein (100 μg rM1 protein) could induce significantly enhanced immune responses, and dose affected the humoral immune response (Table 2) and the cellular immune response (Fig. 3). In terms of serum antibody, mucosal antibody, and specific IFN-γ detected by ELISPOT, only the DNA prime + 100 μg M1 boost immune group and corresponding protein alone groups had significant differences compared with control mice. Only the prime-boost group was completely protected against homologous H9N2 viruses and partially cross-protected against the heterosubtypic PR8 virus. This is probably due to the immune response of the 100 μg M1 boost immune group had been induced sufficiently to stimulate the host and produce better protection. Moreover, 10 μg and 1 μg M1 boost immune group can only detect weak antibody and cellular immune responses, and only provide a insufficient response to resist a lethal homologous virus challenge.

To study the immune mechanism of M1 protein, we synthesized an overlapping peptides library spanning the entire M1 protein. BALB/c (H - 2 d) mice were immunized with the DNA prime-100 μg M1 intranasal boost strategy, then splenic lymphocytes were separated to identigy M1 T cell epitopes using ELISPOT technology. Only peptide M1_11-25_ (VLSIIPSGPLKAEIA) could stimulate T cells to secrete IFN-γ. We also observed that CD4^+^ T cells could be stimulated strongly to secrete IFN-γ, but that CD8^+^ T cells were stimulated weakly by this peptide (Fig. 7).We hypothesized that the M1_11-25_ peptide was an immunodominant M1-specific CD4^+^ T cell epitope,, which could have certain effects speeding up elimination of the virus^40^.

In our study, we only identified immunodominant epitopes, but could not exclude the existence of other M1-specific T cell epitope. Changing the peptide library design or implementing other immune schemes may identify other specific peptides. The immunodominant epitope we identified here could be useful for the study of the mechanisms and functions of influenza virus T cell epitopes. This epitope can also be applied for research and development towards an influenza vaccine immunity test (e.g., as a stimulus) and a universal vaccine (e.g., epitope-based vaccine).

In this study, our focus was the relatively conserved M1 protein as an influenza virus vaccine target. Recombinant M1 protein can join plasmid DNA vaccines to induce mice to produce a cross-protection immune response which could not only protect from a lethal dose of homologous virus challenge, but also provide part of protection against the heterosubtypic virus challenge. The prime-boost strategy can enhance cellular and humoral immune responses, and in our study this strategy showed the most potent cross-protection efficacy. However, the immunogenicity of M1 recombinant proteins needs to be improved to achieve a useful vaccine in the future.

## Materials and Methods

### Viruses, Mice and Cells

Influenza viruses used in this study included a mouse-adapted A/Chicken/Jiangsu/11/2002 (H9N2) and A/PR/8/34(PR8) (H1N1). The H9N2 viruses were used in a biosafety level 3 containment facility in the Shanghai Institute of Biological Products (SIBP). Specific-pathogen-free female BALB/c mice (6–8 weeks old) were purchased and bred in the Animal Resource Center at SIBP as described in our previous studies^12^. All experiments involving animals were approved by the Animal Care Committee of SIBP, in accordance with animal ethics guidelines of the Chinese National Health and Medical Research Council (NHMRC). 293 T and MDCK cells were cultured in Dulbecco’s Modified Eagle Medium (DMEM) containing 10% fetal bovine serum (FBS).

### Vaccine preparation

Plasmid pCAGGSP7/M1(p7/M1) was constructed by cloning PCR products of M gene from the A/Chicken/Jiangsu/7/2002 (H9N2) influenza virus strain into the eukaryotic expression vector pCAGGSP7(p7), as described previously^41^.

Recombinant matrix 1 protein was produced in *Escherichia coli* (*E. coli*) as described previously^12^. The soluble recombinant M1 protein in cell lysate supernatant was purified by affinity chromatography using a nickel-charged sepharose affinity column (Qiagen) according to the manufacturer’s instructions.

### Immunization and challenge

BALB/c mice (n-152, female, 6-8 weeks old) were randomly divided into 8 groups with 19 mice per group. Mice in group A were immunized with one dose of DNA followed by one dose of 100 μg M1 protein; mice in group B were immunized with one dose of 100 μg DNA vaccine followed by one dose of 10 μg M1 protein; mice in group C were immunized with one dose of 100 μg DNA vaccine followed by one dose of 1 μg M1 protein; mice in group D were immunized with 100 μg M1 protein only; mice in group E were immunized with 10 μg M1 protein only; mice in group F were immunized with 1 μg M1 protein only; mice in group G were immunized with 100 μg DNA vaccine only; mice in group H were unimmunized as a negative control; Mice in group D, E, F, G and H were set up as controls. For the DNA vaccine immunization, 50 μl (1 μg/μl) pCAGGSP7/M1 plasmid were applied to both quadriceps femoris muscles. Immunization was followed immediately by electroporation of the injected area^42^. For protein immunization, mice were anesthetized and immunized intranasally with 20 μl of phosphate-buffered saline (PBS) containing a dose of 100, 10 or 1 μg M1 protein, respectively. The time interval between immunizations was two weeks. Two weeks after the last immunization, the mice were anesthetized and challenged with 20 μl of a viral suspension containing 20 × LD_50_ of influenza virus A/Chicken/Jiangsu/7/2002(H9N2) or 5 × LD_50_ A/PR/8/34 (H1N1) by intranasal drip, respectively.

### Sample collection

Nasal washed and BLAF were collected and used for an IgA antibody assays as described in our previous studies^43^. Three days after the challenge, nine mice from each group were randomly taken for sample collection and used for a virus titers assay, and the remaining 10 mice per group were monitored for survival and weight loss, as described in our previous studies^44^. Mice that lost 30% or more of their bodyweight were humanely anaesthetized with chloroform.

### Antibody (Ab) assays

Sera were collected from the blood and used for IgG antibody assays. Specific IgG or IgA antibodies were detected by an ELISA assay as described in our previous studies^12^. Endpoint titres of antigen-specific antibodies were expressed as reciprocal log_2_ titres of the last dilution that showed > means + 2 × SD of background levels. ELISA Ab titers were expressed as the highest serum dilution giving a positive reaction^45^.

### Analyses of lung cytokines

BALF were analyzed for levels of IFN-γ, IL-2 and IL-4 by ELISA using kit assays obtained from Dakewe Biotech Co., Ltd. The absorbance was measured at a wavelength of 450 nm using a microplate reader. The amounts of the cytokines in tested samples were determined using a standard curve, and values were expressed as the mean ± SD.

### IFN-γ ELISPOT assay

The ELISPOT assay described in our previous studies^12^ was used to detect H-2^d^-restricted M1 epitope-specific T cells following stimulation with synthetic peptides. Spleen cells were isolated from mice 2 weeks following the last immunization. Cytokine producing cells were identified as purple spots, and read using an ELISPOT reader (Bioreader4000, Bio-sys, Germany). T cell precursor frequencies for each peptide or pool were based on the total number of cells and number of spot forming cells (SFCs) per well (average of 3 wells). Medium back-grounds were consistently<10 SFC per10^6^ splenocytes.

### Virus titrations

BALF diluted 10-fold serially starting from a dilution of 1:10, then were used to inoculate in MDCK cells at 37 °C for 2 days, so as to examine the cytopathic effect. The virus titre of each specimen, expressed as 50% tissue culture infection dose (TCID_50_), was calculated by the Reed-Muench method. The virus titre in each experimental group was represented as the mean ± SD of the virus titer per ml of specimens from three mice in each group^46^.

### M1 Peptides

A library of overlapping (staggered by 5-6aa) 15-mer peptides spanning the entire M1 protein (A/Chicken/Jiangsu/7/2002(H9N2)) were synthesized by GL Biochem Ltd (shanghai). Purity of these peptides were assessed by mass spectrometry and was >90% purity. The 25-member M1 library was resuspended at 1 mg/ml of each peptide in DMEM and divided into 10 pools of 5 peptides each according to a 5 × 5 matrix. Each pool was used as a stimulus for ELISPOT assay.

### CD4^+^T/CD8^+^T cell depletion for ELISPOT assays

Immunomagnetic cell separation using MACs microbeads (Miltenyi Biotec) was used to separate as the method for separation of CD4^**+**^T/CD8^**+**^T cells, resulting in 90% depletion in 30 min. Depletion was conducted as described by the manufacturer (Mouse CD4^+^T Cell Isolation Kit II and/or Mouse CD8a^+^T Cell Isolation Kit II). Briefly, added 10 μl of biotin-antibody cocktail were added per 10^7^ cells, mixed well and incubated for 10 min at 2–8 °C. Then added 20 μl of anti-biotin microbeads per 10^7^ total cells, mixed well and incubated for 15 min at 2–8 °C. The cell/bead mixture was then put into the Magnetic Activated cell sorting (MACS) Column inside the magnetic field of a suitable MACS Separator, discarded flow-through containing unlabeled cells. Then removed column from the separator and placed it on a suitable collection tube, immediately flushed out the magnetically labeled cells by firmly pushing the plunger into the column for further use in the ELISPOT assays.

### Statistics

Comparisons of experimental groups were evaluated by Student’s t-test; P-values less than 0.05 were considered statistically significant. Survival rates of mice in experimental and control groups were compared using Fisher’s exact test.

## Supplementary information

Dataset 1.
