# Supplementary material for: Immunization with DNA prime-subunit protein boost strategy based on influenza H9N2 virus conserved matrix protein M1 and its epitope screening
Source: Sci Rep. 2020 Mar 5;10:4144. doi: 10.1038/s41598-020-60783-z (PMC7057951; doi:10.1038/s41598-020-60783-z)
Supplement: Supplementary file 1 — Dataset 1. [file 41598_2020_60783_MOESM1_ESM.doc]

**Immunization with DNA prime-subunit protein boost strategy based on influenza H9N2 virus conserved matrix protein M1 and its epitope screening**

Fen Liu^a, #^, Xueliang Wang^a ,c, #^, Mei Zheng^a,*^, Feifei Xiong^a^, Xueying Liu^a^, Linting Zhou^a^, Wensong Tan^b^, Ze Chen^a,*^

**^a^** Shanghai Institute of Biological Products, Shanghai 200052, China.

^b^ State Key Laboratory of Bioreactor Engineering, East China University of Science and Technology, Shanghai 200237, China.

**^c^** Department of Molecular Biology, Shanghai Centre for Clinical Laboratory, Shanghai, China.

**Materials and Methods**

Three days after the challenge, the trachea and lungs were collected. After high-speed homogenization of bronchopulmonary tissues with a total of 2ml of PBS containing 0.1% BSA, the supernatant was centrifuged for detection of lung virus titer.

**qPCR:** RNA extraction of lung homogenates was prepared by the TRIzol (Invitrogen, US) homogenization method according to the manufacturer’s recommendations. The purity and concentration of the extracted RNA samples were examined by UV5Nano Spectrophotometer (Mettler Toledo, Swiss). Reverse transcription of RNA and qPCR was performed by PrimeScript™ RT-PCR kit (Takara, Japan) and iQ™ SYBR® Green Supermix kit (Bio-Red, US), respectively. Real-time qPCR was carried out on an ABI PRISM 7300 Sequence Detector (Applied Biosystems, USA). The primers used were as follows: for H9N2 HA: forward, 5’ - ACGGGAAAGGATGTTTTGA; reverse, 5’- GCGACAGTCGAATAAATGG, for PR8 HA: forward, 5’ - ACACCCCTGGGAGCTATAA; reverse, 5’- CCTTCAATAAAACCGGCAA, and for 18s rRNA: forward, 5’- AGGGGAGAGCGGGTAAG; reverse, 5’- GGACAGGACTAGGC. 18s rRNA was used as an internal control. For data analysis, the value of HA/18s rRNA was used to calculate fold change. All samples were performed in triplicate, and the final data represented the mean of at least three individual experiments. The virus titer in each experimental group was represented by the mean ± SD of the virus titer per ml of specimens from three mice in each group^46^.

**Plaque assay**: At these time points, the lungs were harvested and homogenized using a BeadBlaster 24 homogenizer (Benchmark), and viral lung titers were measured by plaquing lung homogenates on MDCK cells. MDCK cells in six-well plates were inoculated with 10-fold dilutions of the lung homogenates in DMEM medium containing 5ug/ml TPCK trypsin (Sigma, US). After incubated at 37°C for 1 h, cells then were then replaced with an agar overlay (DMEM, 5ug/ml TPCK trypsin). Cells inoculated without lung homogenates and overlaid with agar were set up as controls. Cells were stained with crystal violet solution to visualize plaques on 72h after virus inoculation.

The virus titer in each experimental group was represented by the mean ± SD of the virus titer per ml of specimens from three mice in each group.

Table S1 Protection against lethal avian influenza H9N2 virus challenge in mice by DNA prime intranasal protein boost strategy based on M1 vaccine

| Group | Immunogen | Lung virus titer (*10^5^ pfu/ml) ^a^ | | |  | HA/18s relative fold ^a^ | | |
| --- | --- | --- | --- | --- | --- | --- | --- | --- |
|  |  | 3 days | 5 days | 7 days |  | 3 days | 5 days | 7 days |
| A | 100μg DNA+100μg M1 | 3.23±0.55 | 0.93±0.61 ^b,c^ | 0.08±0.07 |  | 1.12±0.04 | 0.26±0.27 ^b^ | 0.05±0.03 ^b^ |
| B | 100μg DNA+10μg M1 | 2.83±0.42 | 3.57±1.15 | 0.18±0.09 |  | 0.33±0.19 | 2.24±0.93 | 0.57±0.37 ^b,d^ |
| C | 100μg DNA+1μg M1 | 2.80±0.20 | 4.30±0.70 | 0.19±0.01 |  | 0.47±0.28 | 2.29±0.11 | 1.08±0.46 |
| D | 100μg M1 | 2.9±0.92 | 3.17±0.47 | 0.06±0.04 |  | 0.50±0.27 | 1.05±0.34 ^b^ | 0.34±0.41 ^b^ |
| E | 10μg M1 | 2.57±0.15 | 3.5±0.82 | 0.28±0.05 |  | 0.09±0.07 | 1.24±1.04 | 2.45±2.21 |
| F | 1μg M1 | 2.13±0.15 | 2.7±0.10 | 0.24±0.14 |  | 0.47±0.35 | 1.17±1.16 | 1.52±2.54 |
| G | 100μg DNA | 2.83±0.81 | 4.97±1.79 | 0.36±0.41 |  | 0.07±0.39 | 4.43±2.66 | 1.87±0.80 |
| H | control | 1.63±047 | 2.20±0.17 | 0.22±0.10 |  | 1.00±0.42 | 4.0±3.00 | 2.33±3.12 |

One hundred and fifty-two BALB/c mice were randomly divided into groups of eight. Nineteen mice in each group were immunized as described above. Two weeks post-vaccination, mice were challenged with a lethal dose (20×LD_50_) of avian influenza H9N2 virus (homologous virus). Mouse lung homogenate from three mice in each group were collected 3^rd^, 5^th^ and 7^th^ days post-infection for titration of lung virus and qPCR, respectively.

^a^ Results are expressed as mean ± SD of tested mice in each group

^b^ Significant difference compared to the mice in control groups(p < 0.05).

^c^ Significant difference compared to the mice in D groups(p < 0.05).

^d^ Significant difference compared to the mice in E groups(p < 0.05).
